# The Beautiful Sunshine

**Published:** 1870-01

**Authors:** 


### THE BEAUTIFUL SUNSHINE.

Persons who have been at Rome will remember that the charge for a southside room is nearly double that for one of northern exposure. This is the result of a practical fact impressed upon the minds of the people from the observation of centuries, that sunshine is healthful; and yet very few New Yorkers seemed to have arrived to that height of intelligence. Read over the advertisements any day for "furnished rooms," and the indispensable requisite, next to "a high-stoop brown-stone, west side," is that it shall be a front room; it may front a pig-pen or a plank-yard, a stable or a steamery, all the same, only if it is a "front room," to overlook the street! as if we would die if we couldn't see something; as if there was nothing to do but to sit at the window and gaze at the passers-by by the hour.

A New York merchant noticed that all his book-keepers became consumptive in a few years, and died. One day it occurred to him it might be the result of their occupying a room where the sunshine never entered, in consequence of high walls. Next day he gave his clerk a sunshiny room, and never had a consumptive book-keeper afterward.

Another New York merchant placed his son on a beautiful improved farm in Illinois. The best upper room of the house overlooked the prairie. Three years later that son returned to New York an invalid—the cough, the hectic, the death! On close inquiry, he stated to his physician that he always found his clothing damp and moldy. "Did the sun ever shine in your room?" "No, sir; it was on the north side of the house."

